# Non-adherence to Statin Treatment: A Narrative Review of Causes, Clinical Impact, and Preventive Strategies

**DOI:** 10.7759/cureus.108870

**Published:** 2026-05-14

**Authors:** Tomasz Mróz, Jeremi Morka, Michal Sobczak, Aleksandra Morajko

**Affiliations:** 1 Faculty of Medicine, University of Opole, Opole, POL

**Keywords:** cardiovascular risk, lipid disorders, non-adherence, preventive medicine, statins

## Abstract

This narrative review examines the determinants and clinical consequences of non-adherence to statin (HMG-CoA (3-hydroxy-3-methylglutaryl coenzyme A) reductase inhibitor) therapy and evaluates evidence-based strategies for improving long-term medication adherence in patients with hyperlipidemia. It is important to note that prescribing treatment alone will not be effective without sufficiently prolonged adherence to prescribed pharmacotherapy. Non-adherence to prescribed treatment represents a significant clinical challenge. In the following article, we have identified the main causes of the phenomenon, its clinical effects, and summarized reports on possible prevention of the phenomenon. The aim of this narrative literature review was to evaluate factors that may negatively impact long-term adherence to statin therapy in patients with hyperlipidemia, and to identify interventions that could improve medication adherence and reduce discontinuation rates during statin therapy. A search of the bibliographic databases, including PubMed and Google Scholar, was conducted using combinations of the terms “statin,” “statin therapy,” “adherence,” “non-adherence,” “compliance,” “persistence,” “discontinuation,” “hyperlipidaemia,” “lipid disorders,” “cardiovascular risk,” “statin intolerance,” and “adherence interventions.” No formal lower date restriction was applied; however, recent clinically relevant studies and systematic reviews were prioritized, while seminal older studies were included where necessary for clinical and conceptual context. Additional relevant publications were identified through manual review of reference lists from selected studies. Articles without direct relevance to statin therapy, animal studies, and publications with limited relevance to the objectives of this review were excluded. Because this was a narrative review, no formal quality assessment or meta-analysis was performed. The evidence was synthesized narratively and organized into three main themes: determinants of statin non-adherence, clinical implications of non-adherence, and strategies to improve adherence. A total of 22 articles were used as the basis for this study, allowing us to provide practical insights into the reasons for non-adherence to statin treatment. A review of the literature indicates that economic factors, psychopathological factors, and cognitive factors all contribute to statin non-adherence. A patient’s failure to comprehend the purpose of prescribed treatment, in conjunction with a lack of awareness regarding the consequences of non-adherence, also plays a crucial role in non-adherence to lipid-lowering treatment. It is recommended that patients be made aware of the potential risks associated with non-adherence to statin treatment. Research highlights the crucial role of the physician in building confidence in the provided treatment. It is essential to educate patients from the outset of the treatment about their medication and to provide ongoing support during follow-up visits. This approach may help to reduce the number of patients who discontinue their statin and other prescribed medication treatment.

## Introduction and background

Statins are a widely prescribed class of medications that effectively lower blood cholesterol levels, particularly low-density lipoprotein cholesterol (LDL-C), commonly referred to as “bad cholesterol.” They function by inhibiting the enzyme HMG-CoA (3-hydroxy-3-methylglutaryl coenzyme A) reductase, a key enzyme in the liver’s cholesterol biosynthesis pathway. By reducing LDL-C concentrations, statins help prevent cholesterol accumulation in arterial walls, thereby significantly lowering the risk of atherosclerosis, myocardial infarction, stroke, and other major cardiovascular events [1,2].

In addition to their primary lipid-lowering action, statins exert a range of pleiotropic effects, including anti-inflammatory activity, improvement of endothelial function, and stabilization of atherosclerotic plaques [3]. These additional properties further contribute to their cardiovascular protective benefits. Statins rank among the most extensively studied pharmacological agents, with numerous large-scale clinical trials [4] consistently demonstrating their efficacy in reducing cardiovascular morbidity and overall mortality.

Given that cardiovascular disease remains the leading cause of death globally [5], a trend expected to continue due to aging populations, the high rates of statin non-adherence are particularly concerning [6,7]. The following review explores the multifactorial determinants of non-adherence and outlines potential strategies to improve long-term patient non-adherence with statin therapy.

## Review

Etiology: determinants of statin non-adherence

Despite statins' established efficacy in reducing cardiovascular risk, a 2025 meta-analysis of 76 studies with nearly 6 million participants reports pooled good adherence (defined as ≥80% medication possession ratio) at only 62.4% over a median of 24 months, dropping to 57.5% in primary prevention, underscoring an urgent need to dissect the etiology of non-adherence [8].

The reasons for poor adherence to statin therapy are multifactorial, but psychopathological elements play a crucial role. These factors influence patients’ perceptions, behaviors, and interactions with healthcare providers. A meta-analysis by Grenard et al. (2011) of 31 studies involving 18,245 participants found a significant association between depression and medication non-adherence (r = -0.16, 95% CI: -0.20 to -0.11, p < 0.001), with depressed patients having 1.76 times the odds of being non-adherent compared to non-depressed patients, corresponding to a 16% risk difference [9]. This association was consistent across various chronic diseases, including hyperlipidemia and hypertension [9]. Another study involving 244 adults with stage C heart failure found that depressed patients were 2.3 times more likely to self-report poor medication adherence than non-depressed patients (OR: 2.26, 95% CI: 1.26-4.07, p = 0.006), though this association was not confirmed by objective adherence measures (p = 0.56) [10].

Anxiety, particularly when related to health, can manifest as excessive worry about potential side effects. In the context of statins, patients may catastrophize common, mild symptoms (e.g., muscle aches), attribute unrelated somatic complaints to the medication, or stop treatment preemptively. The ASCOT-LLA trial, which included 10,180 patients randomly assigned to atorvastatin 10 mg or placebo, found that muscle-related adverse events were reported at similar rates during the blinded phase (2.03% vs. 2.00% per year; HR: 1.03, 95% CI: 0.88-1.21; p = 0.72), whereas during the non-blinded phase, they were reported more frequently among statin users than non-users (1.26% vs. 1.00% per year; HR: 1.41, 95% CI: 1.10-1.79; p = 0.006), supporting the role of a nocebo effect in perceived statin intolerance. These findings suggest that patients’ expectations and beliefs about statin therapy may influence the perception of side effects [11].

Older adults or those with early cognitive decline may unintentionally forget to take medications or mismanage complex regimens. A cross-sectional study of elderly patients with chronic diseases found that mild cognitive impairment was an independently influential factor of medication non-adherence, with affected patients nearly four times more likely to exhibit poor adherence compared to cognitively intact peers (OR: 3.95, 95% CI: 2.63-5.92, p < 0.001), underscoring the importance of early cognitive screening in the context of long-term pharmacotherapy, including statin treatment [12].

According to Li et al., non-adherence to statin therapy is strongly influenced by psychopathological factors, particularly patients’ beliefs regarding the necessity and safety of their medication [13]. The authors emphasize that low necessity beliefs, where patients doubt the importance of statins for their health, constitute a major determinant of non-adherence, especially in asymptomatic individuals who may not perceive an immediate benefit from treatment. While concerns about adverse effects are common, Li et al. found that these concerns alone do not independently predict non-adherence, suggesting that the perceived lack of relevance of statin therapy is a more significant barrier [13]. Commonly reported reasons for non-adherence to long-term drug therapy, including statins, are outlined in Table 1.

**Table 1:** The causes of statin non-adherence divided into factors related to the patient and factors unrelated to the patient.

Category	Aspect	Examples of reasons
Patient-related factors	Cognitive factors	Forgetfulness, lack of knowledge about the condition, psychological issues (e.g., depression and anxiety)
	Behavioral factors	Low motivation, fear of side effects
Non-patient-related factors	Socioeconomic-related factors	Low income, poor social support, low health literacy, unstable housing or job, cost of treatment, cultural beliefs impacting medicine use
	Health system-related factors	Poor provider, patient communication, lack of access to health services, short consultation times, inconvenient pharmacy hours
	Condition-related factors	Chronic or asymptomatic diseases, mental disorders, complex comorbidities
	Therapy-related factors	Complex treatment regimens, long duration of treatment, side effects, unpleasant route of administration

Clinical implications of statin non-adherence

The most notable implication of statin non-adherence is increased cardiovascular risk. A 2024 systematic review from the Journal of the American Geriatrics Society found 92% higher risk for all-cause mortality (HR: 1.92, 95% CI: 1.52-2.44, nine studies), cardiovascular mortality (HR: 1.63, 95% CI: 1.27-2.10, five studies), and cardiovascular events (HR: 1.31, 95% CI: 1.23-1.39, eight studies). In this study, a noteworthy observation was also made for end-of-life populations where statin discontinuation does not appear to affect 60-day mortality, underlining the importance of an individualized patient approach (risk difference: 3.5%, 90% CI: −3.5 to 10.5) [14].

Another key consideration in the consequences of discontinuing statin treatment is the risk of post-stroke complications. A study involving 479 participants assessed adherence to statins in patients with a previous acute stroke incident (mean age = 58.3 years). Of these, 21.8% were not receiving a statin, 34.9% had poor adherence, 9.1% had fair adherence, and 35.0% had good adherence. Treatment discontinuation occurred in 15.7% of cases. Poor adherence was associated with hypertension, previous stroke, and large-artery atherosclerosis, and was associated with worse functional outcome and stroke recurrence. Significant functional recovery was only observed in patients with good adherence to statins [15].

In contrast, one of the most important complications of statin use is myopathies, which often result in statin discontinuation, thus contributing to adverse cardiovascular outcomes. Myopathy problems rarely occur as a complication of statin use alone, but often occur as a result of the improper combination of statins with multiple drugs or accompanying chronic diseases such as kidney or liver failure [16,17].

Remedial strategies

Several effective interventions aimed at increasing statin adherence have been identified in recent large-scale systematic research. The most consistently supported one is patient education and counseling [18]. Patient knowledge and belief in the importance of regular statins use is cited by Li et al. to be a greater factor in adherence even than lack of side effects [13].

Improving support and communication with patients is a central component of effective statin adherence interventions. Addressing limited awareness of cholesterol risks and the low perceived threat of cardiovascular disease is essential, as understanding the benefits of statin therapy facilitates adherence. Providing clear, accessible information about statin effectiveness and the dangers of untreated cholesterol, including the use of relatable analogies (such as unclogging pipes), helps patients appreciate the importance of treatment. Discussing statin safety, potential side effects, and appropriate responses to adverse effects can further reduce uncertainty, particularly when mild side effects are framed in the context of the more serious risks associated with untreated cardiovascular disease [19].

Establishing trust and fostering collaboration between patients and healthcare professionals is also critical. Poor interactions and distrust are recognized barriers, while encouragement, support, and shared decision-making from healthcare providers promote adherence. Communication should focus on engaging patients in discussions about their concerns, actively listening, and tailoring recommendations to individual needs and lifestyles. Integrating conversations about complementary approaches, such as diet and exercise, and adjusting treatment plans based on patient feedback can build trust and address side effects more effectively. It is worth noting, however, that seeking alternatives before starting statin therapy can be a major driving factor in non-adherence, and focus on those being only complementary to statins should be clearly stated to the patient [20].

Reducing the burden of treatment is another important strategy. Many patients struggle with complex medication regimens, forgetfulness, and competing priorities. Practical solutions, such as informing patients about automatic refills, coordinating refill dates, providing guidance on medication interactions, supporting habit formation, and encouraging family involvement, can simplify medication management and help patients feel more confident in adhering to their statin therapy. Regular follow-up, reminders, and the use of medication packaging or organizational aids further support these efforts [21]. Aforementioned strategies also help with adherence in patients with mild cognitive impairment, where, alongside early mild cognitive impairment identification and treatment, employing them will be beneficial.

A holistic approach that actively supports mental health can also significantly improve statin adherence. Available evidence suggests that interventions fostering positive attitudes toward medication, reducing symptoms of anxiety and depression, and addressing perceived stress are associated with better adherence outcomes [22]. Integrating mental health support, such as counseling, psychoeducation, and regular assessment of psychological well-being, into statin therapy enables patients to manage emotional barriers that might otherwise undermine consistent medication use. Moreover, strategies that build trust, encourage open communication, and provide tailored support for both psychological and practical challenges further empower patients to maintain their treatment regimens. By prioritizing mental health alongside physical health, care teams can create a more supportive environment in which patients are better equipped to adhere to statin therapy over the long term.

## Conclusions

Low adherence to statin therapy is a multifaceted issue, often driven by the intersection of psychological and psychiatric factors. Effectively addressing this problem requires a holistic, patient-centered approach that calls for integration of medical management with psychological support and educational interventions. Psychopathological elements, such as depression, anxiety, cognitive dysfunction, and maladaptive beliefs, may meaningfully affect patients’ ability and willingness to maintain long-term pharmacotherapy. Overcoming these barriers calls for a multidisciplinary approach, including mental health screening, empathetic communication, and straightforward medication regimens. Identifying and addressing the emotional and cognitive obstacles to adherence is essential for improving therapeutic outcomes and reducing cardiovascular risk.

While the proposed strategies largely represent common-sense, low-risk adaptations of established adherence interventions, we must acknowledge their evidence base derives from relatively modest-sized studies, dwarfed by the landmark trials establishing statins' lipid-lowering and mortality benefits. Thus, despite the compelling insights from the current literature, further large-scale research is essential to validate and optimize these psychopathological-targeted approaches for sustained clinical impact.
